# Analgesic effects of lappaconitine in leukemia bone pain in a mouse model

**DOI:** 10.7717/peerj.936

**Published:** 2015-05-07

**Authors:** Xiao-Cui Zhu, Chen-Tao Ge, Pan Wang, Jia-Li Zhang, Yuan-Yang Yu, Cai-Yun Fu

**Affiliations:** Lab of Proteomics & Molecular Enzymology, School of Life Sciences, Zhejiang Sci-Tech University, Hangzhou, China

**Keywords:** Lappaconitine, Leukemia, Bone pain, Analgesia

## Abstract

Bone pain is a common and severe symptom in cancer patients. The present study employed a mouse model of leukemia bone pain by injection K562 cells into tibia of mouse to evaluate the analgesic effects of lappacontine. Our results showed that the lappaconitine treatment at day 15, 17 and 19 could effectively reduce the spontaneous pain scoring values, restore reduced degree in the inclined-plate test induced by injection of K562 cells, as well as restore paw mechanical withdrawal threshold and paw withdrawal thermal latency induced by injection of K562 cells to the normal levels. Additionally, the molecular mechanisms of lappaconitine’s analgesic effects may be related to affect the expression levels of endogenous opioid system genes (POMC, PENK and MOR), as well as apoptosis-related genes (Xiap, Smac, Bim, NF-*κ*B and p53). Our present results indicated that lappaconitine may become a new analgesic agent for leukemia bone pain management.

## Introduction

Bone pain is a common and severe symptom in cancer patients, especially in advanced stage (Jimenez-Andrade et al., 2010; Kane, Hoskin & Bennett, 2015; Mantyh, 2014; Mantyh & Hunt, 2004). Many patients with hematological malignancies (leukemia, lymphoma, myeloma, myelodysplastic syndromes and myeloproliferative neoplasms) frequently experience pain (Niscola et al., 2011). Currently the molecular mechanisms underlying leukemia bone pain are largely unknown. The available pharmacological tools for bone pain analgesia are limited, with unstable efficacy and sometimes adverse side effects (Kane, Hoskin & Bennett, 2015; Mantyh, 2014). Therefore, it is important to search for new therapeutic drugs against leukemia bone pain, especially from traditional Chinese medicine (TCM) agents.

Aconitum (Wu Tou) is a common TCM drug used for analgesia (Singhuber et al., 2009). Lappaconitine is one bioactive component isolated from aconitum sinomonatum nakai with clinical efficacy in chronic pain and inflammation (Ono & Satoh, 1988; Ono & Satoh, 1991; Wang et al., 2009; Wright, 2001), which is the most effective drug presently available for the treatment of malignant tumor and other intractable pain (Wang et al., 2009). Using methods for screening of analgesics, the results obtained from Ono and Satoh showed that the analgesic effects of lappaconitine were generally about 2 to 5 times less potent than those of morphine (Ono & Satoh, 1988; Ono & Satoh, 1989; Ono & Satoh, 1990). The use of lappaconitine reduces pain in liver cancer patients, and can alleviate their dependence on morphine treatment (Chen, Wang & Lin, 1996; Liu, Zhu & Tang, 1987). In addition, lappaconitine shows no addition properties, nor toxicity against nervous system and heart. However, there is no report about the effect of lappaconitine on pain induced by leukemia cells. The present study aimed to examine the potential application of lappaconitine in leukemia bone pain.

## Materials and Methods

### Ethics

The study has been approved by ethic committee of animal research in Hangzhou Normal University. All procedures followed guidelines of animal research in Hangzhou Normal University (permit number: 2014-0023), and the animal pain research guidelines of International Association for the Study of Pain (IASP).

### Animal model

80 female ICR mouse were provided by animal center in Hangzhou normal university with free access to food and water (12/12 h light cycle, 5 mouse in each cage). The animals were randomly assigned into 4 groups: normal group (*n* = 20), normal saline group (*n* = 20), bone pain group (K562 cell transplanted group) (*n* = 20), and bone pain with lappaconitine treatment group (K562 cells+lappaconitine group) (*n* = 20).

The leukemia bone pain model was built through injection of chronic myeloid leukemia K562 cells into tibial bone marrow cavity directly. Briefly, the animals were anesthetized with halothane and placed in supine position. The amount of 4 × 10^5^(1 × 10^7^/ml, 40 μl) K562 leukemia cancer cells or same volume of saline were infused into the left tibia marrow cavity through a microsyringe. The puncture was sealed by the medical glue and the wound area was smeared with erythromycin eye ointment in accordance with the protocols of aseptic operation. The animals were let for recovery in a warm place before sending back to home cage.

### Lappaconitine treatment

The lappaconitine (Maya Medical Equipment Co. Ltd, Shanghai, China) were given through intraperitoneal (i.p.) injection at 4 mg/kg on day 15, day 17, and day 19 after cancer cell transplantation.

### Pain behavior scoring

For spontaneous pain scoring, the mouse were placed in 30 cm × 40 cm × 40 cm (height, length, width) open field for spontaneous activity recording and evaluation. Score 0: free movement, same limb activity of treated limb as the control limb. Score 1: slight limp movement of the treated limb. Score 2: moderate limp. Score 3: severe limp movement. Score 4: loss of ground touch for the affected limb, as described previously (Mao-Ying et al., 2006).

For inclined plate test to assess the muscular strength and the proprioception according to previous methods (Ou et al., 2011), the mouse were placed on the inclined plane, in vertical position to the long axis. If the mouse could keep balance for 5 s, the inclined degree will be increased by 2° with an initial angle of 30°. The loss-balance degree for the inclined plate was recorded.

For the paw mechanical withdrawal threshold (PMWT) measurement, von Frey hair (Stoelting, Wood dale, Illinois, US) was used as described previously (Kim & Chung, 1992). A series of von Frey hair (0.16 g, 0.4 g, 0.6 g 1.0 g, 1.4 g, 2.0 g, 4.0 g, 6.0 g) were employed to stimulate the left foot center according to up-and-down approach, starting from 0.4 g. The non-response was recorded as “O” and response as “X”. 50% PMWT (g) = 10 ^log(*f*)+*kδ*^. *δ* is taken as 0.224 in present study, and *k* is taken from the scale based on “O” and “X” recordings.

For paw withdrawal thermal latency (PWTL) measurement, a YLS-21A cold-hot plate was used. The mouse were placed on 55° hot plate and the pain response (limb licking or lifting) latencies were recorded. Each mouse was tested for 5 times with 10 min interval. The average of three median values was recorded.

For tail illumination pain test of tail-flick latency, the mouse tail was illuminated under 30W light and the latency for tail flick was recorded. Each mouse was tested for 5 times with 10 min interval. The average of three median values was recorded.

### PT-PCR assay

The total RNA was extracted from the tibial bone marrow cells using TRlzol Reagent (Invitrogen Corp., Carlsbad, California, USA) according to the manufacturer’s instructions. Two µg of total RNA in each group was reverse transcribed into cDNA in a final volume of 50 µl as in previous reports (Fu et al., 2014; Zhang et al., 2014). The gene of GAPDH was selected as an endogemous internal control gene and a non-template reaction was included as negative control for each experiment. The PCR primers and Tm value of each gene were summarized in Table 1. The PCR conditions were as follows: 1 cycle of 95 °C for 3 min followed by 30 cycles of 95 °C for 30 s, 45–53 °C (depending on the Tm value of each gene) for 30 s and 72 °C for 30 s; and 1 cycle of 72 °C for 10 min.

**Table 1:** Sequences of forward and reverse primers of indicated target genes and the Tm value of primers.

Gene name	Sequence	Tm (°C)
MOR(F)	ATCCTCTCTTCTGCCATTGGT	58.01
MOR(R)	TGAAGGCGAAGATGAAGACA	55.75
POMC(F)	AGATTCAAGAGGGAGCTGGA	57.80
POMC(R)	CTTCTCGGAGGTCATGAAGC	59.85
PENK(F)	AACAGGATGAGAGCCACTTGC	59.97
PENK(R)	CTTCATCGGAGGGCAGAGACT	61.92
Bim(F)	TGTGTGTAAACATAATGCGGG	56.06
Bim(R)	TGAGGTGAAGTCACAGGACAC	59.97
Xiap(F)	AGTGGGGCACCACATGTTAT	57.80
Xiap(R)	CGGAAACAGTGCTGTTAGCA	57.80
Smac(F)	GCGGTTCCTATTGCTCAGAA	57.80
Smac(R)	GGATGTGATTCCTGGCAGTT	57.80
p53(F)	CCTCCCCAGCATCTTATCCG	61.90
p53(R)	CACAAACACGAACCTCAAA	53.25
NF-*κ*B (RELA) (F)	GTTCACAGACCTGGCATCTGT	59.97
NF-*κ*B (RELA) (R)	GAGAAGTCCATGTCCGCAATG	59.97
NK1R-Tr(F)	GGGCCACAAGACCATCTACA	60.30
NK1R-Tr(R)	AAGTTAGCTGCAGTCCCCAC	60.30
GAPDH(F)	AGGAGCGAGATCCCTCCAAAAT	61.94
GAPDH(R)	GTGATGGCATGGACTGTGGT	59.85

### Statistics

Each group consisted of 20 mice. The data were represented as mean ± SEM and analyzed with a one-way analysis of variance (ANOVA) followed by the Dunnett’s test (two-sided) for post hoc comparisons on all time course studies. A probability level of less than 0.05 was considered significant.

## Results

### The bone pain model on body weight

**Figure 1:**
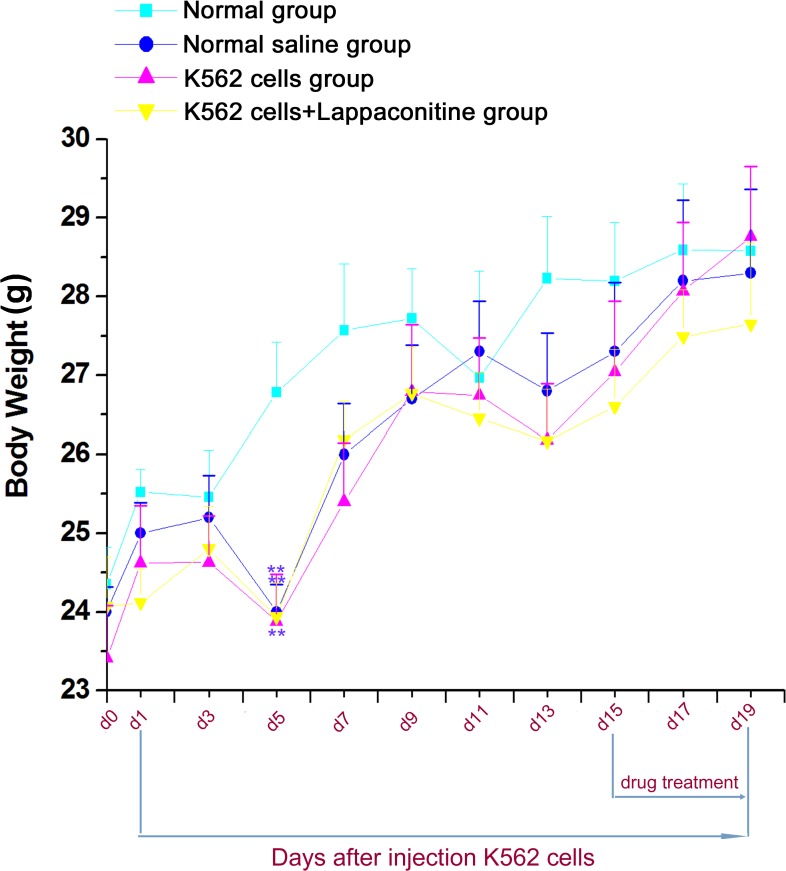
Body weight changes in the four groups. Data are expressed as means ± SEM. ^∗∗^*P* < 0.01, compared with the normal group on each corresponding day.

There was no clear difference between control and the bone pain group until 5 days after K562 cell transplantation. The then, bone pain group mouse began to exhibit reduced spontaneous activity, and decreased ground touch for the affected limb. The body weights in four groups had no differences except for day 5, in which the body weight of the inoculative groups was decreased markedly, indicating that the inoculation technology may affect the appetite in the first few days, as shown in Fig. 1.

### Pain behavior scoring

**Figure 2:**
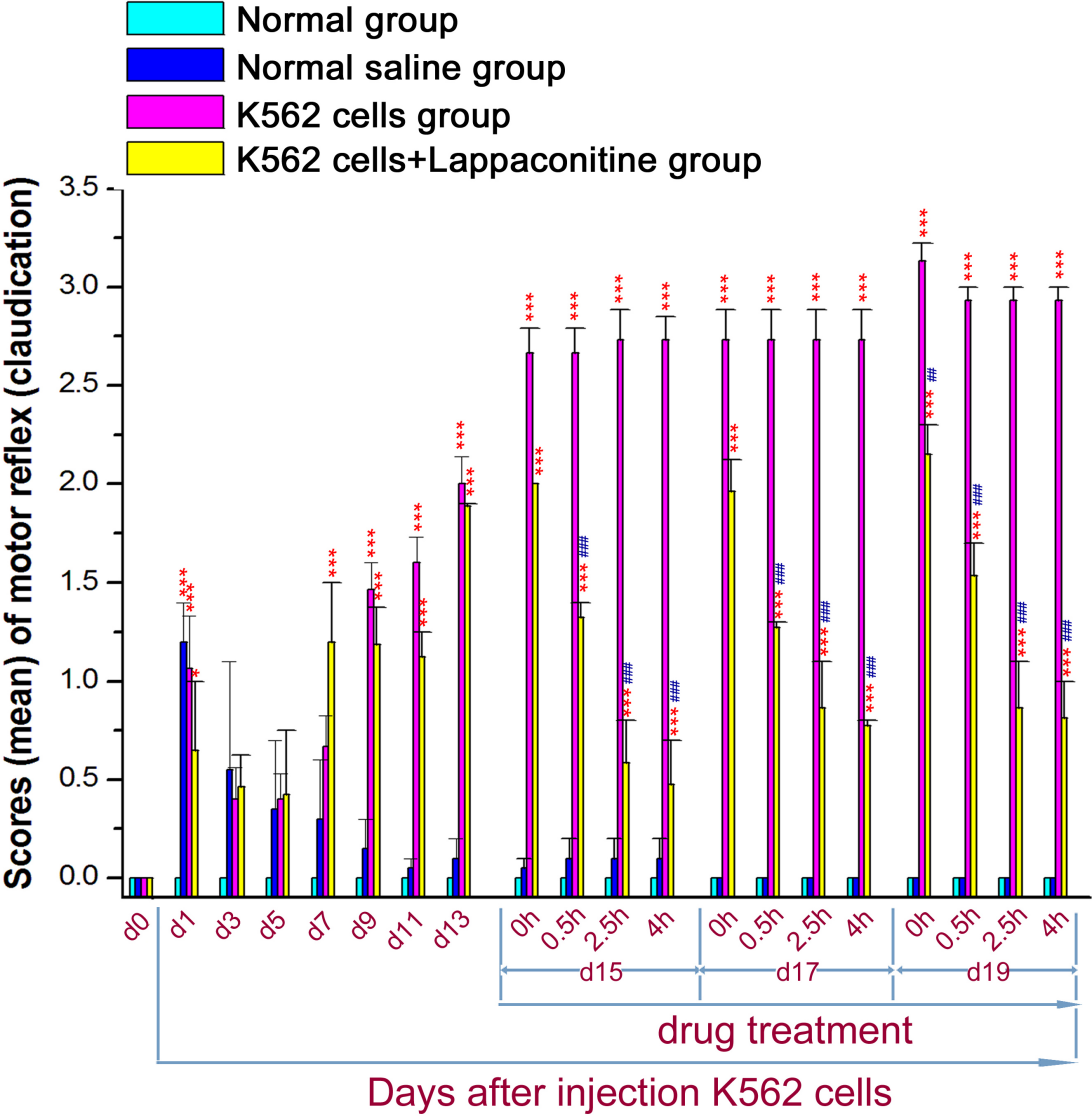
Spontaneous pain scoring in the four groups. Data are expressed as means ± SEM. ^∗^*P* < 0.05, ^∗∗∗^*P* < 0.001, compared with the control groups on each corresponding day. ^##^*P* < 0.01, ^###^*P* < 0.001, compared with the K562 cells group on each corresponding day.

For spontaneous pain scoring, all mice in each group moved in a normal way in prior to the manipulation. The infusion technique caused limp movement just on day 1 in all the infusion groups. The K562 cell transplanted group exhibited spontaneous pain which was began at 7–9 days after injection of K562 cells, lasting for the rest of the experiment; the treatment with lappaconitine at 4 mg/kg on day 15, 17 and 19 reduced the spontaneous pain significantly compared to the untreated K562 group, but did not alleviate the pain completely (Fig. 2, ^∗^*P* < 0.05, ^∗∗∗^*P* < 0.001, compared with the control groups; ^##^*P* < 0.01, ^###^*P* < 0.001, compared with the K562 cells group).

**Figure 3:**
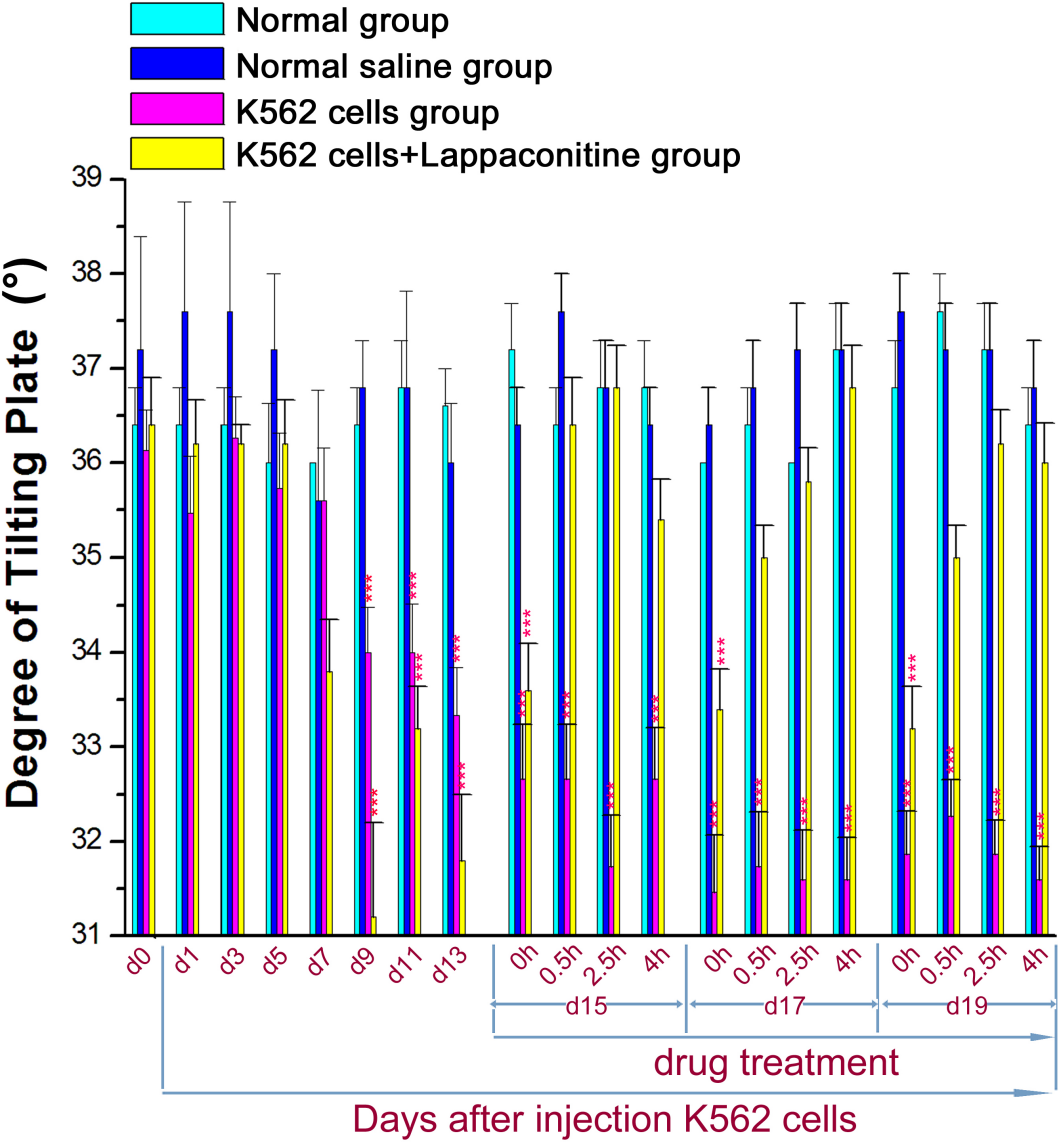
Inclined-plate test in the four groups. Data are expressed as means ±SEM. No significant deficits in the hind-limb motor function were found in normal group and normal saline (NS) group, while the degree was decreased markedly in the K562 cells transplanted group began at day 9. ^∗∗∗^*P* < 0.001 among all groups compared with the control groups.

Inclined-plate test was employed to evaluate the muscle functioning and the body balance behavior. The degree that mice could maintain balance was reduced markedly after 9 days in the K562 cells transplanted group, which was restored by lappaconitine treatment on day 15, 17 and 19 (Fig. 3, ^∗∗∗^*P* < 0.001 compared with the control groups).

**Figure 4:**
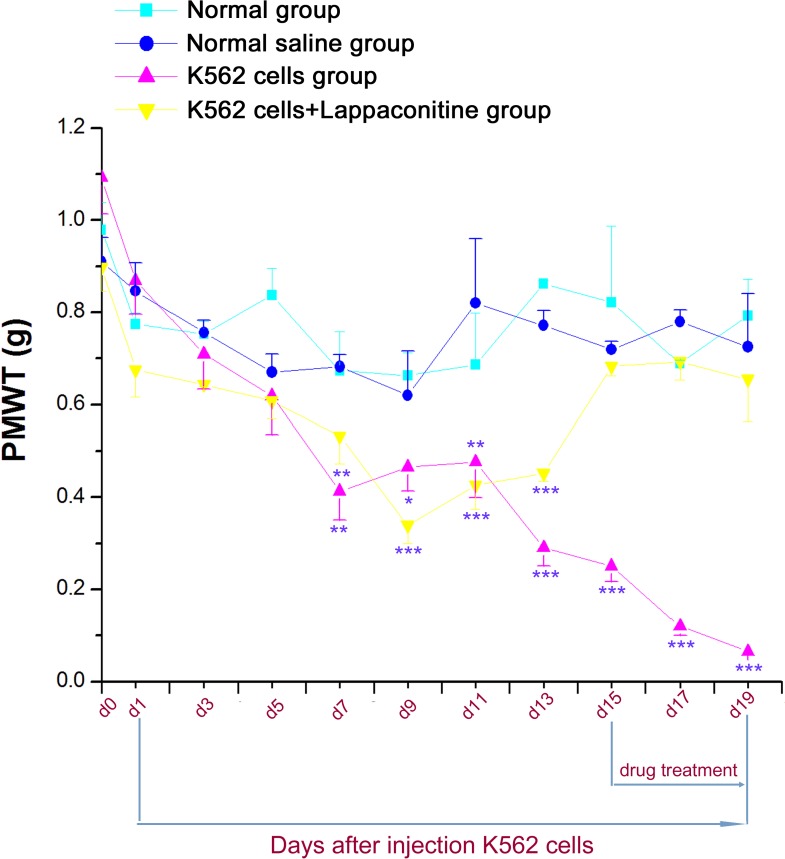
Paw mechanical withdrawal threshold (PMWT) in the four groups. Data are expressed as means ± SEM. ^∗^*P* < 0.05, ^∗∗^*P* < 0.01, ^∗∗∗^*P* < 0.001, compared with control groups on each corresponding day.

**Figure 5:**
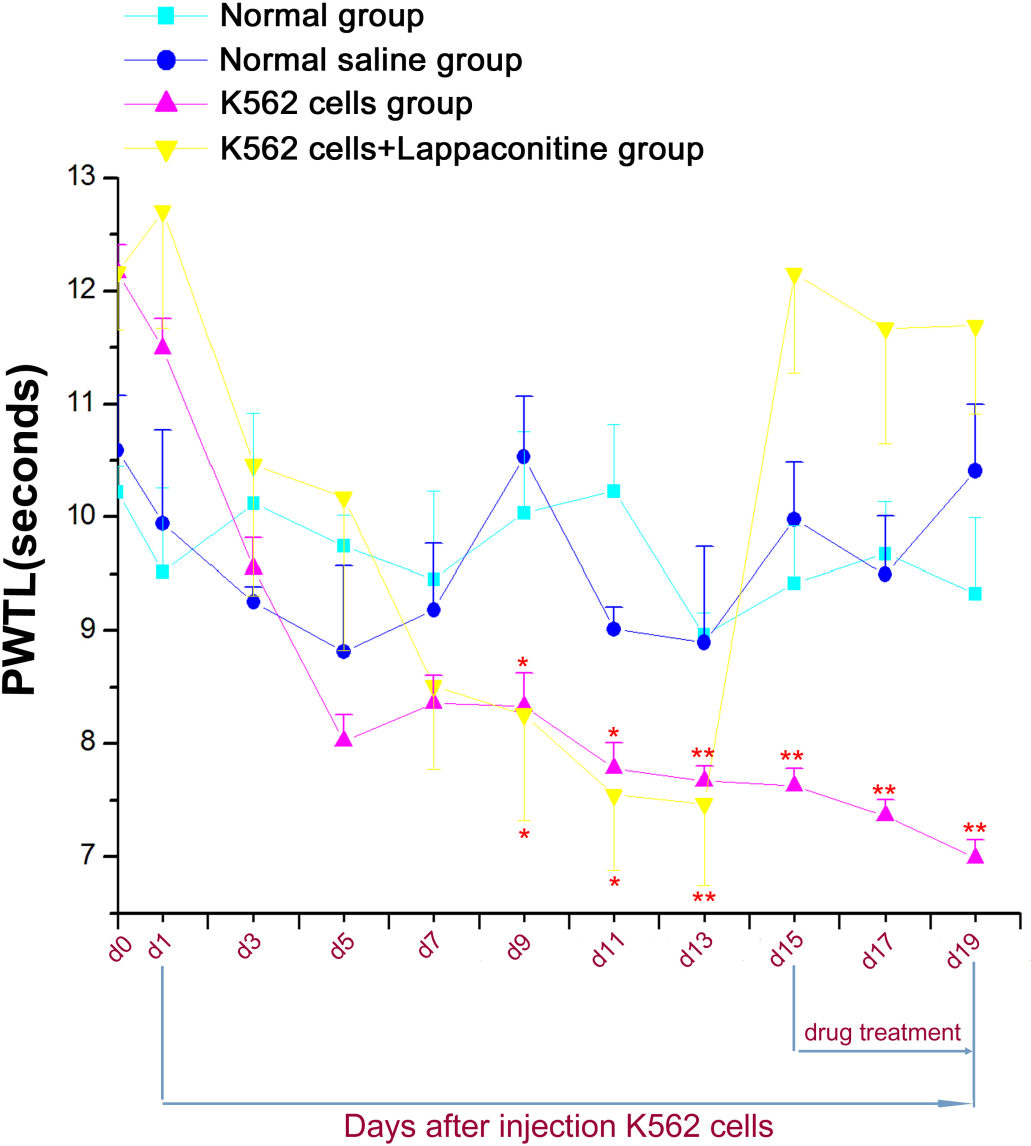
Paw withdrawal thermal latency (PWTL) in the four groups. Data are expressed as means ± SEM. ^∗^*P* < 0.05, ^∗∗^*P* < 0.01, compared with control groups on each corresponding day.

The von Frey hair tests and the hot plate tests were used to assess the sensitivity of mouse paws to mechanical (Fig. 4) and thermal (Fig. 5) stimulation, respectively. Our results showed that both the paw mechanical withdrawal threshold (PMWT) and the paw withdrawal thermal latency (PWTL) were decreased significantly after 7–9 days in the K562 cells transplanted group and lappaconitine treatment on day 15, 17 and 19 could also restored both the PWTL and PMWT values to the levels of control groups, respectively which was similar to the previous pain behavior tests (Figs. 4 and 5, ^*^P < 0.05, ^∗∗^*P* < 0.01, ^∗∗∗^*P* < 0.001, compared with control groups).

**Figure 6:**
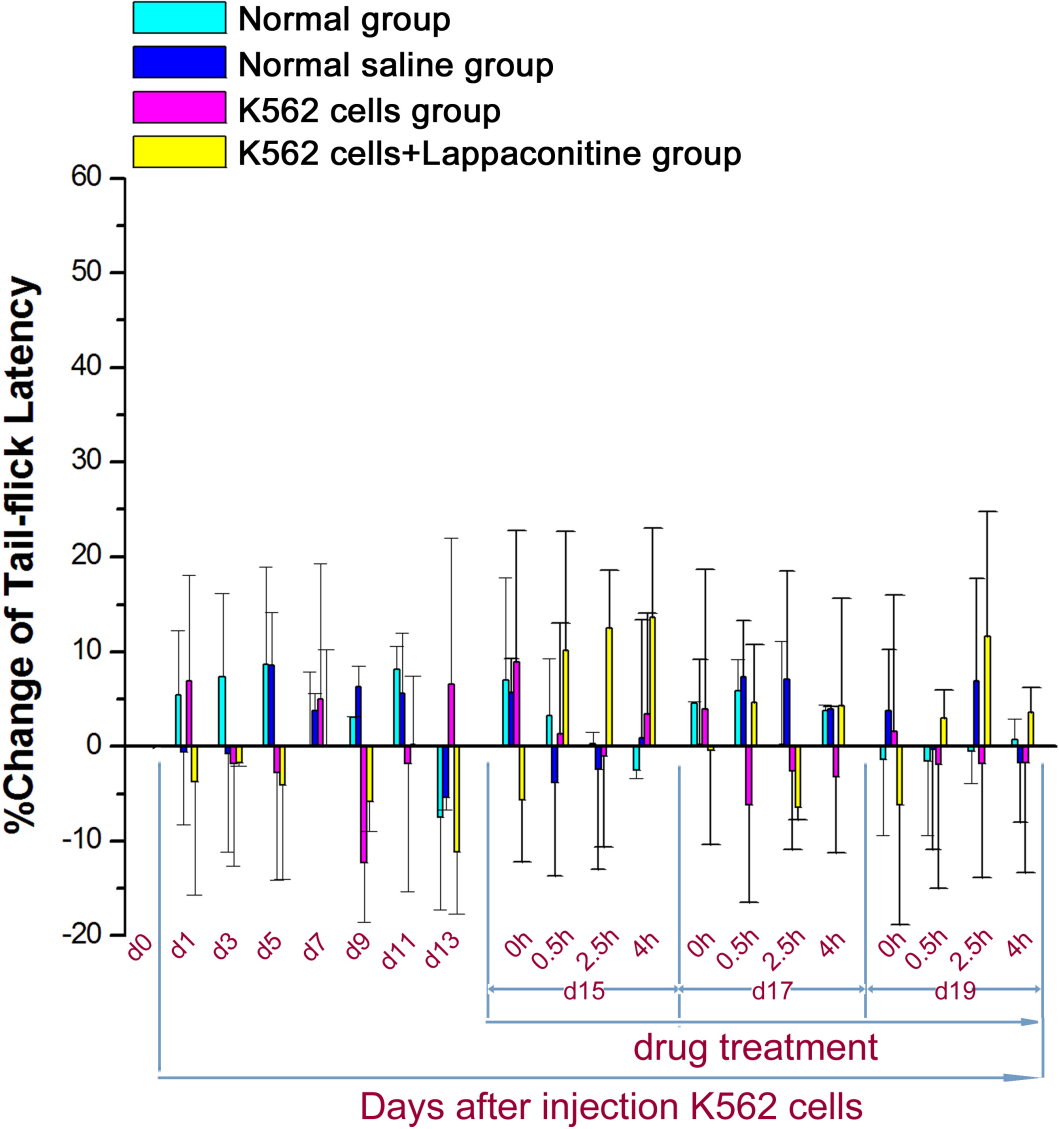
Tail illumination pain test of tail-flick latency in the four groups. Data are expressed as means ± SEM. There were no significant changes in the K562 cell transplanted group.

For tail illumination pain test of tail-flick latency, no clear differences were observed among the four groups mentioned above, suggesting that this kind of pain behavior might be deficit in present animal model of cancer bone pain (Fig. 6).

### Changes of the mRNA expressions of indicated target genes

**Figure 7:**
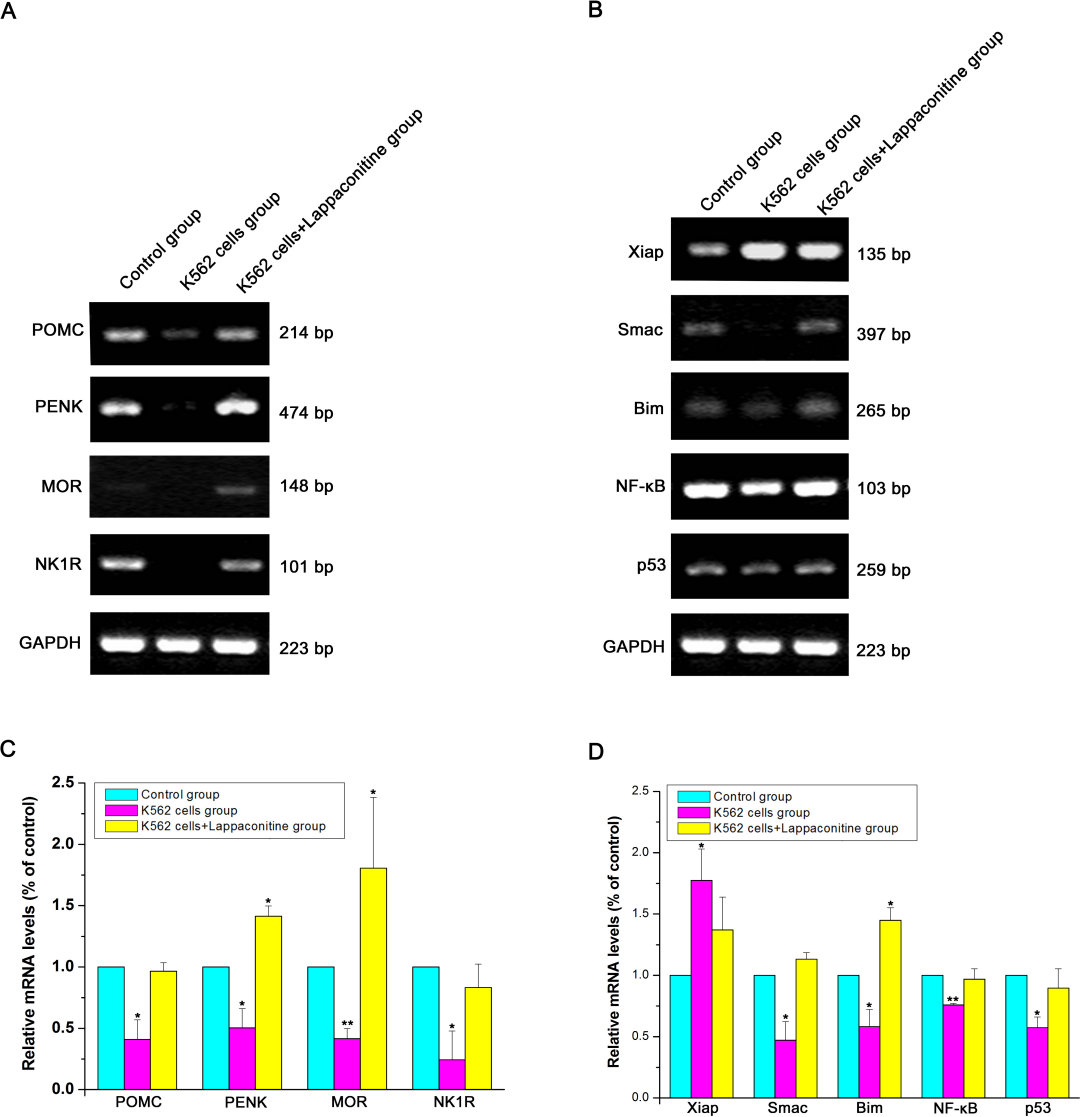
Expression of indicated target genes at mRNA level. The density of the labeled bands for amplified products of target genes as well as the housekeeping GAPDH gene was shown in each group. Relative intensity for each gene compared with GAPDH was measured using Image Quant software. Figures 7C and 7D are statistical analysis of relative intensity for each gene in Figs. 7A and 7B, respectively, compared with each corresponding control gene. ^∗∗^*P* < 0.01, ^∗∗∗^*P* < 0.001, compared with control groups of each corresponding gene.

Several target genes of endogenous opioid system (POMC, PENK and MOR), apoptosis-related genes (Xiap, Smac, Bim, NF-*κ*B and p53), and the neurokinin receptor 1 (NK1R) gene were detected to explore the potential molecular mechanisms involved in our leukemia bone cancer pain. In these indicated target genes, our results showed that the expression levels of these target genes were decreased markedly except for the Xiap gene that was increased markedly in the K562 cells transplanted group, while lappaconitine treatment could return their expression levels to the control group levels or raised their expression levels markedly (Fig. 7, ^∗∗^*P* < 0.01, ^∗∗∗^*P* < 0.001, compared with the control group of each gene).

## Discussion

The number of cancer patients is growing, and 60–90% of late stage patients suffered from cancer pain, including 30% of patients with severe lasting pain (Buga & Sarria, 2012; Kane, Hoskin & Bennett, 2015; Mantyh, 2014). Bone pain is a common symptom in cancer pain caused by cancer metastasis to bone tissue (Jimenez-Andrade et al., 2010; Sabino & Mantyh, 2005). With the increase of survival rates of cancer patients in recent years, the life quality of the patients is still challenged by the presence of bone pain.

In recent years, the animal model of cancer bone pain is built for pharmacological screening of new therapeutic agents. For instance, NCTC2472 bone cancers cells, MRMT-1 breast cancer cells, Lewis lung cancer cells, melanoma cancer cells, and prostate cancer cells have been employed to set up the animal model (De Ciantis et al., 2010; Donovan-Rodriguez, Dickenson & Urch, 2005; Dore-Savard et al., 2010; Mao-Ying et al., 2006; Medhurst et al., 2002; Zhang & Lao, 2012). In our present study, our results showed that the values of pain behavior scoring were changed significantly on day 7 to day 9 in the K562 cells transplanted group, which is consistent with the behavioral changes of bone cancer pain in animal models transplanted with solid tumor cells (De Ciantis et al., 2010; Dore-Savard et al., 2010; Mao-Ying et al., 2006; Medhurst et al., 2002; Zhang & Lao, 2012). To our knowledge, this is the first to employ leukemia cancer cells to build the bone pain animal model of leukemia, with minimal invasion in the surgery.

Lappaconitine, a diterpenoid alkaloid extracted from the roots of Aconitum Sinomontanum Nakai, has been used as analgesia, local anesthetic, as well as antifebric and anti-inflammatory agents for decades (Guo & Tang, 1990; Guo & Tang, 1991; Ono & Satoh, 1988; Ou et al., 2011; Wang et al., 2009; Wright, 2001). The use of lappaconitine reduces pain in liver cancer patients, and can alleviate their dependence on morphine treatment (Chen, Wang & Lin, 1996; Liu, Zhu & Tang, 1987), with no toxicity against nervous system and heart (Heubach & Schule, 1998). Our present study showed that lappaconitine could relive the pain behaviors induced by the injection of leukemia K562 cells into the tibial bone marrow cavity, which maybe related to the changes the expression levels of endogenous opioid genes, as well as apoptosis-related genes. The present study is the first to investigate the efficiency of lappaconitine in leukemia bone pain, and highlighted its potential as analgesic agents in bone pain of other cancers.

## Supplemental Information

10.7717/peerj.936/supp-1 Supplemental Information 1 Raw data

10.7717/peerj.936/supp-2 Supplemental Information 2 Raw data

10.7717/peerj.936/supp-3 Supplemental Information 3 Raw data Raw data for Fig 3

10.7717/peerj.936/supp-4 Supplemental Information 4 Raw data Raw data for Fig 4

10.7717/peerj.936/supp-5 Supplemental Information 5 Raw data Raw data for Fig 5

10.7717/peerj.936/supp-6 Supplemental Information 6 Raw data Raw data for Fig 6
